# Supplementary material for: Content-rich biological network constructed by mining PubMed abstracts
Source: BMC Bioinformatics. 2004 Oct 8;5:147. doi: 10.1186/1471-2105-5-147 (PMC528731; doi:10.1186/1471-2105-5-147)
Supplement: Additional File 2 — The original results of the above study (non-essential files are deleted to keep the file size under the limit set by BMC bioinformatics). [file 1471-2105-5-147-S2.bz2 › chilibotAdditionalFile2/dip30/590ID9195885E10962/html/NTHA_NTHB.html]

 


 **NTHA** and **NTHB** 
  
Found 122 abstracts in PubMed, retrieved 30.  
 

 What does Google say? 
 PDF only 
| .edu only 

---

**Interactive relationship** (e.g. stimulation, inhibition, etc)

**Neutral relationship**- Competitive binding studies demonstrate that MeCN preferentially binds over ROH, suggesting that nitriles would be capable of displacing the H 2 O coordinated to the iron site of NHase  [ **NTHB** ]   [ **NTHA** ] .  Ref: 12236756 J Am Chem Soc, 2002
- Reported herein is a five coordinate model compound Fe III S 2 Me2 N 3 Et, Pr containing Fe III in an environment resembling that of NHase  [ **NTHB** ]   [ **NTHA** ] , which reversibly binds a variety of nitriles, alcohols, amines, and thiocyanate.  Ref: 12236756 J Am Chem Soc, 2002

**Non-interactive relationship** (e.g. studied together, co-existance, homology, etc.)

- ABN, AHN and AFN were converted by various microorganisms belonging to different bacterial genera e.g. Pseudomonas or Rhodococcus expressing either nitrilase  [ **NTHB** ]   [ **NTHA** ]  or nitrile hydratase activities.  Ref: 12845494 Appl Microbiol Biotechnol< MedlineTA>Appl Microbiol Biotechnol, 2002
- A rapid and sensitive fluorometric assay method for the determination of nitrilase  [ **NTHB** ]   [ **NTHA** ]  activity.  Ref: 12549980 Biotechnol Appl Biochem< MedlineTA>Biotechnol Appl Biochem, 2002
- The primer pairs, NHCo1 NHCo2 and NHFe1 NHFe2 yield PCR products corresponding to a partial coding sequence of cobalt and iron NHase  [ **NTHB** ]   [ **NTHA** ]  genes, respectively.  Ref: 11682195 FEMS Microbiol Lett, 2001
- This suggests that the active site for cyanide hydratase and nitrilase  [ **NTHB** ]   [ **NTHA** ]  activity in the protein is the same.  Ref: 12725921 FEMS Microbiol Lett, 2003
- A series of iron complexes based on the pentadentate ligand bis 2 methyl 2 mercaptopropyl 1 thia diazacyclononane, bmmp TASN 2, have been synthesized and characterized as models of iron containing nitrile hydratase NHase  [ **NTHB** ]   [ **NTHA** ] .  Ref: 12844310 Inorg Chem< MedlineTA>Inorg Chem, 2003
- Here we show yeast glutamine dependent NAD synthetase Qns1 requires both the nitrilase  [ **NTHB** ]   [ **NTHA** ]  related active site residues and the NAD synthetase active site residues for function in vivo.  Ref: 12771147 J Biol Chem< MedlineTA>J Biol Chem, 2003
- The purified former four mutant enzymes showed neither enzymatic activity nor the maximum absorption at 410 nm which was detected in the wild type H NHase  [ **NTHB** ]   [ **NTHA** ] .  Ref: 12121763 J Inorg Biochem, 2002
- These data indicate that the nitrilase  [ **NTHB** ]   [ **NTHA** ]  related domain in Qns1 is the fourth independently evolved GAT domain to have been identified in nature.  Ref: 12771147 J Biol Chem< MedlineTA>J Biol Chem, 2002
- The recombinant enzyme shows highest nitrilase  [ **NTHB** ]   [ **NTHA** ]  activity with benzonitrile.  Ref: 12725921 FEMS Microbiol Lett, 2003
- Three dimensional models for other amidases belonging to the nitrilase  [ **NTHB** ]   [ **NTHA** ]  superfamily also predicted Cys Glu Lys catalytic triads.  Ref: 11955282 Biochem J, 2002
- This nitrilase  [ **NTHB** ]   [ **NTHA** ]  gene was over expressed in Escherichia coli, generating a microorganism that efficiently and regioselectively catalyzes the conversion of aliphatic dinitriles to cyanocarboxylic acids.  Ref: 12655453 Appl Microbiol Biotechnol, 2003
- The nitrilase  [ **NTHB** ]   [ **NTHA** ]  AtNIT1 from Arabidopsis thaliana was overexpressed in Escherichia coli with an N terminal His6 tag and purified by zinc chelate affinity chromatography in a single step almost to homogeneity in a 68% yield with a specific activity of 34.1 U.mg 1.  Ref: 11856328 Eur J Biochem, 2002
- Collectively, these data suggest that the elevated levels of IAM in seeds and germinating seedlings result from nitrilase  [ **NTHB** ]   [ **NTHA** ]  action on indole 3 acetonitrile, a metabolite produced in the plants presumably from glucobrassicin turnover.  Ref: 12430025 Planta, 2002
- The attempted conversion of the 2 acetoxynitriles to almost stoichiometric amounts of the corresponding 2 acetoxycarboxylic acids was finally achieved by using either a recombinant E. coli strain that highly overexpressed the nitrilase  [ **NTHB** ]   [ **NTHA** ]  gene from the pseudomonad or the purified enzyme derived from this strain.  Ref: 12845494 Appl Microbiol Biotechnol< MedlineTA>Appl Microbiol Biotechnol, 2002
- These cysteine residues i.e. alpha C102, alpha C105 and alpha C107 in the high molecular mass nitrile hydratase H NHase  [ **NTHB** ]   [ **NTHA** ]  of Rhodococcus rhodochrous J1 were replaced with alanine by site directed mutagenesis using the R. rhodochrous ATCC12674 host vector system, and the resultant transformants were investigated.  Ref: 12121763 J Inorg Biochem, 2002
- And the activity of NHase  [ **NTHB** ]   [ **NTHA** ]  in solution of 45% acrylamide was just about half of that in solution of 5% acrylamide.  Ref: 12148289 Sheng Wu Gong Cheng Xue Bao, 2002
- Taken together, these results suggest that a regulatory loop appears to exist by which sulphate deficiency, through an increase in glucobrassicin turnover and nitrilase  [ **NTHB** ]   [ **NTHA** ]  3 accumulation, initiates the production of extra auxin leading to increased root growth and branching, thus allowing the root system to penetrate new areas of soil effectively to gain access to fresh supplies of sulphur.  Ref: 11967096 Plant J, 2002
- Arabidopsis thaliana expresses four nitrilases, three of which NIT1, NIT2 and NIT3 are able to convert indole 3 acetonitrile to indole 3 acetic acid IAA, the plant growth hormone, while the isozyme NIT4 is a beta cyano l alanine hydratase nitrilase  [ **NTHB** ]   [ **NTHA** ] .  Ref: 11967096 Plant J, 2002
- The nitrilase  [ **NTHB** ]   [ **NTHA** ]  superfamily classification, structure and function.  Ref: 11380987 Genome Biol, 2001
- or =60% identity to enzymes of the beta alanine synthase nitrilase  [ **NTHB** ]   [ **NTHA** ]  family.  Ref: 11673419 J Bacteriol, 2001
- The aliphatic amidase from Pseudomonas aeruginosa belongs to the nitrilase  [ **NTHB** ]   [ **NTHA** ]  superfamily, and Cys 166 is the nucleophile of the catalytic mechanism.  Ref: 11955282 Biochem J, 2002
- The characterization of the enzyme at the molecular level has provided new insights into how the molecular structure determines the enzyme function, and how the regulatory system controls the expression of the enzyme genes to improve the enzyme and the NHase  [ **NTHB** ]   [ **NTHA** ]  dependent process.  Ref: 11893064 Chem Rec, 2001
- Both nitrilase  [ **NTHB** ]   [ **NTHA** ]  polypeptide and nitrilase  [ **NTHB** ]   [ **NTHA** ]  activity were also induced by sulphur starvation.  Ref: 11967096 Plant J, 2002
- A polymerase chain reaction PCR protocol was developed for the specific detection of genes coding nitrile hydratase NHase  [ **NTHB** ]   [ **NTHA** ] .  Ref: 11682195 FEMS Microbiol Lett, 2001
- Thus, recombinant AtNIT1 resembles in its properties the native enzyme and the nitrilase  [ **NTHB** ]   [ **NTHA** ]  from Brassica napus.  Ref: 11856328 Eur J Biochem, 2002
- The proposed method is more sensitive than the existing methods for the determination of nitrilase  [ **NTHB** ]   [ **NTHA** ]  activity.  Ref: 12549980 Biotechnol Appl Biochem< MedlineTA>Biotechnol Appl Biochem, 2002
- Earlier we discovered that glutamine dependent NAD synthetases contain N terminal domains that are members of the nitrilase  [ **NTHB** ]   [ **NTHA** ]  superfamily and hypothesized that these domains function as glutamine amidotransferases GATs for the associated synthetases.  Ref: 12771147 J Biol Chem< MedlineTA>J Biol Chem, 2002
- The mRNA expression of key enzymes involved in the synthesis, conjugate hydrolysis, accumulation and basipetal transport of auxin, namely indole 3 glycerol phosphate synthase, nitrilase  [ **NTHB** ]   [ **NTHA** ] , IAA amino acid hydrolase, chalcone synthase and PIN1 as an essential component of the basipetal IAA carrier, was investigated by reverse transcription polymerase chain reaction.  Ref: 12624772 Planta, 2003
- We report a novel heterologous system for the expression of catalytically active iron containing NI1 NHase  [ **NTHB** ]   [ **NTHA** ]  in Escherichia coli, involving coexpression with the E. coli GroES and GroEL chaperones.  Ref: 12729727 Protein Expr Purif, 2003
- A potential role for NIT2 in breaking seed dormancy in A. thaliana by low temperatures stratification was ruled out, although NIT2 was the predominantly expressed nitrilase  [ **NTHB** ]   [ **NTHA** ]  isoform in developing embryos and in germinating seeds, as judged from an analysis of beta glucuronidase reporter gene expression under the control of the promoters of the four isogenes.  Ref: 11525507 Planta, 2001
- The presence of a nitrilase  [ **NTHB** ]   [ **NTHA** ]  gene family and nitrilase  [ **NTHB** ]   [ **NTHA** ]  activity in extracts from T. majus suggests that PAA might be synthesized by the nitrilase  [ **NTHB** ]   [ **NTHA** ]  pathway using benzylglucosinolate as precursor.  Ref: 12060252 Physiol Plant< MedlineTA>Physiol Plant, 2001
- No other nitrilase  [ **NTHB** ]   [ **NTHA** ]  genes respond to sulphur supply deficiency.  Ref: 11967096 Plant J, 2002
- Eukaryotic NAD synthetase Qns1 contains an essential, obligate intramolecular thiol glutamine amidotransferase domain related to nitrilase  [ **NTHB** ]   [ **NTHA** ] .  Ref: 12771147 J Biol Chem< MedlineTA>J Biol Chem, 2002
- Whereas AguA has no structural relationship to any known C N hydrolases, AguB is a protein of the nitrilase  [ **NTHB** ]   [ **NTHA** ]  family that performs thiol assisted catalysis.  Ref: 12634339 Microbiology, 2003
- Purification, cloning, sequencing and over expression in Escherichia coli of a regioselective aliphatic nitrilase  [ **NTHB** ]   [ **NTHA** ]  from Acidovorax facilis 72W.  Ref: 12655453 Appl Microbiol Biotechnol, 2003
- Several plasmids were constructed to express the enzyme under the control of the lac promoter or its own promoter, together with or without nitrilase  [ **NTHB** ]   [ **NTHA** ]  and a possible regulatory protein that is present in the wild type genome.  Ref: 12651117 Protein Expr Purif, 2003
- A rapid, simple and sensitive fluorometric assay method for the determination of nitrilase  [ **NTHB** ]   [ **NTHA** ]  activity is described.  Ref: 12549980 Biotechnol Appl Biochem< MedlineTA>Biotechnol Appl Biochem, 2003
- Recently, we defined the nitrilase  [ **NTHB** ]   [ **NTHA** ]  superfamily as consisting of 12 families of amidases, N acyltransferases and presumptive amidases, in addition to the family of plant and bacterial nitrilases for which the superfamily was named.  Ref: 12504683 Curr Opin Struct Biol, 2002
- Microbial nitrile hydratase NHase  [ **NTHB** ]   [ **NTHA** ]  has great potential as a catalyst in organic chemical processing because the enzyme can convert nitriles to the corresponding higher value amides under mild conditions, and has now been applied to the industrial productions of acrylamide and nicotinamide.  Ref: 11893064 Chem Rec, 2001
- In order to suppress unwanted side reactions, biotransformations were performed with recombinant Escherichia coli strains that heterologously expressed nitrilase  [ **NTHB** ]   [ **NTHA** ]  activities originating from Pseudomonas, Rhodococcus, or Synechocystis strains.  Ref: 12845494 Appl Microbiol Biotechnol< MedlineTA>Appl Microbiol Biotechnol, 2001
- Using the PCR method, both types of iron and cobalt NHase  [ **NTHB** ]   [ **NTHA** ]  encoding genes were detected in DNA from pure cultures and soil samples.  Ref: 11682195 FEMS Microbiol Lett, 2001
- This is the first evidence of cyanide hydratase having nitrilase  [ **NTHB** ]   [ **NTHA** ]  activity.  Ref: 12725921 FEMS Microbiol Lett, 2003
- These data show for the first time that, when it is contained in a ligand environment similar to that of NHase  [ **NTHB** ]   [ **NTHA** ] , Fe III is capable of forming a stable complex with nitriles.  Ref: 12236756 J Am Chem Soc, 2002
- Purified genomic DNA from bacterial strains or directly from soil can serve as the target for the PCR, thus affording a simple and rapid method for screening NHase  [ **NTHB** ]   [ **NTHA** ]  genes.  Ref: 11682195 FEMS Microbiol Lett, 2001
- Earlier, we noted a perfect correlation between presence of a domain related to nitrilase  [ **NTHB** ]   [ **NTHA** ]  and glutamine dependence and then proved, in an accompanying paper, that the nitrilase  [ **NTHB** ]   [ **NTHA** ]  related domain is an essential, obligate intramolecular, thiol dependent glutamine amidotransferase in the yeast NAD synthetase, Qns1.  Ref: 12777395 J Biol Chem< MedlineTA>J Biol Chem, 2001
- A regioselective aliphatic nitrilase  [ **NTHB** ]   [ **NTHA** ]  from Acidovorax facilis 72W was purified and characterized, and the corresponding gene was cloned and sequenced.  Ref: 12655453 Appl Microbiol Biotechnol, 2003
- This paper studied the effects of concentration of cells, temperature, pH value, concentrations of acrylonitrile and acrylamide on the deactivation of Nhase  [ **NTHB** ]   [ **NTHA** ] , as well as the related enzyme de active kinetics.  Ref: 12148289 Sheng Wu Gong Cheng Xue Bao, 2002
- Structural analysis revealed in both NADsyn1 and NADsyn2 a domain required for NAD synthesis from ammonia and in only NADsyn1 an additional carbon nitrogen hydrolase domain shared with enzymes of the nitrilase  [ **NTHB** ]   [ **NTHA** ]  family that cleave nitriles as well as amides to produce the corresponding acids and ammonia.  Ref: 12547821 J Biol Chem, 2003
- After study on the relation of temperature and the reaction speed, It was found that the activation energy of the hydration of NHase  [ **NTHB** ]   [ **NTHA** ]  was 65.57 kJ.mol 1.  Ref: 12148289 Sheng Wu Gong Cheng Xue Bao, 2002
- The spectroscopic and structural properties of bmmp TASN FeNO BPh 4 1 bmmp TASN = bis 2 methyl 2 mercaptopropyl 1 thia diazacyclononane have been determined and are compared with the nitric oxide inactivated form of iron containing nitrile hydratase, NHase  [ **NTHB** ]   [ **NTHA** ]  dark.  Ref: 11874334 Inorg Chem, 2002
- A model of amidase was built by comparative modelling using the crystal structure of the worm nitrilase  [ **NTHB** ]   [ **NTHA** ]  fragile histidine triad fusion protein NitFhit.  Ref: 11955282 Biochem J, 2002
- The nu NO stretching frequency of 1856 cm 1 and the Mössbauer parameters, delta = 0.06 mm s and DeltaE q = 1.75 mm s, compare favorably with those of NHase  [ **NTHB** ]   [ **NTHA** ]  dark.  Ref: 11874334 Inorg Chem, 2002
- Site directed mutagenesis of the F. lateritium cyanide hydratase gene indicates that mutations leading to a loss of cyanide hydratase activity also lead to a loss of nitrilase  [ **NTHB** ]   [ **NTHA** ]  activity.  Ref: 12725921 FEMS Microbiol Lett, 2003
- The cyanide hydratase protein produced at a high level in Escherichia coli shows a low but significant nitrilase  [ **NTHB** ]   [ **NTHA** ]  activity with acetonitrile, propionitrile and benzonitrile.  Ref: 12725921 FEMS Microbiol Lett, 2003
- The cyanide hydratase enzyme of Fusarium lateritium also has nitrilase  [ **NTHB** ]   [ **NTHA** ]  activity.  Ref: 12725921 FEMS Microbiol Lett, 2003
- Catalysis in the nitrilase  [ **NTHB** ]   [ **NTHA** ]  superfamily.  Ref: 12504683 Curr Opin Struct Biol, 2002
- In addition, we report a rapid and convenient spectrophotometric method to monitor the activity of NI1 NHase  [ **NTHB** ]   [ **NTHA** ]  during purification.  Ref: 12729727 Protein Expr Purif, 2003
- Based upon these findings, these three cysteine residues were found to be essential for the active expression of H NHase  [ **NTHB** ]   [ **NTHA** ] .  Ref: 12121763 J Inorg Biochem, 2002
- Rapid and specific identification of nitrile hydratase NHase  [ **NTHB** ]   [ **NTHA** ]  encoding genes in soil samples by polymerase chain reaction.  Ref: 11682195 FEMS Microbiol Lett, 2001
- Recombinant nitrilase  [ **NTHB** ]   [ **NTHA** ]  isoforms and 3, known to synthesize IAA from indole 3 acetonitrile, were shown to produce significant amounts of IAM in vitro as a second end product of the reaction besides IAA.  Ref: 12430025 Planta, 2002
- Two of them were up regulated genes encoding casein kinase II alpha subunit and glycine rich protein Grp, and the other two down regulated genes encoding nitrilase  [ **NTHB** ]   [ **NTHA** ]  associated protein and 18S small subnit ribosomal RNA gene respectively.  Ref: 12561473 Yi Chuan Xue Bao, 2002
- The inactive, nitrosyl bound form of Fe type nitrile hydratase NHase  [ **NTHB** ]   [ **NTHA** ]  contains two active site cysteine residues that are post translationally modified to sulfenate SO and sulfinate SO2 ligands.  Ref: 12430453 Chem Commun Camb , 2002
- Sodium dodecyl sulfate polyacrylamide gel electrophoresis SDS PAGE for the cell free extracts of each mutant transformant revealed that four mutant transformants i.e. alpha C105A, alpha C107A, alpha C102A C105A and alpha C105A C107A showed predominant alpha and beta subunit protein bands with a mobility identical to those of the native H NHase  [ **NTHB** ]   [ **NTHA** ] , while three mutant transformants i.e. alpha C102A, alpha C102A C107A and alpha C102A C105A C107A did not produce the corresponding proteins.  Ref: 12121763 J Inorg Biochem, 2002
- Recent experimental results confirm the essentiality of the catalytic triad residues and specify the biochemical functions of additional branches and sub branches of the nitrilase  [ **NTHB** ]   [ **NTHA** ]  superfamily.  Ref: 12504683 Curr Opin Struct Biol, 2002
- The nitrilase  [ **NTHB** ]   [ **NTHA** ]  superfamily consists of thiol enzymes involved in natural product biosynthesis and post translational modification in plants, animals, fungi and certain prokaryotes.  Ref: 11380987 Genome Biol, 2001
- The nitrilase  [ **NTHB** ]   [ **NTHA** ]  activity is sufficient for growth of the recombinant strain on acetonitrile, propionitrile or benzonitrile as the sole source of nitrogen.  Ref: 12725921 FEMS Microbiol Lett, 2003
- A nitrilase  [ **NTHB** ]   [ **NTHA** ]  like protein from Arabidopsis thaliana NLP1 was expressed in Escherichia coli as a His 6 tagged protein and purified to apparent homogeneity by Ni 2 chelate affinity chromatography.  Ref: 12435743 J Biol Chem, 2003
- Thus, glutamine dependent NAD synthetase may be classified as a possible glutamine amidase in the nitrilase  [ **NTHB** ]   [ **NTHA** ]  family.  Ref: 12547821 J Biol Chem, 2003
- Enzymatic characterization of the recombinant Arabidopsis thaliana nitrilase  [ **NTHB** ]   [ **NTHA** ]  subfamily encoded by the NIT2 NIT1 NIT3 gene cluster.  Ref: 11525507 Planta, 2001
- Three of the nitrilase  [ **NTHB** ]   [ **NTHA** ]  isoenzymes of Arabidopsis thaliana L. Heynh.  Ref: 11525507 Planta, 2001
- It was also found that the concentration of acrylonitrile had little effect on the stability of NHase  [ **NTHB** ]   [ **NTHA** ] .  Ref: 12148289 Sheng Wu Gong Cheng Xue Bao, 2002
- Nitrile hydratase NHase  [ **NTHB** ]   [ **NTHA** ]  is an iron containing metalloenzyme that converts nitriles to amides.  Ref: 12236756 J Am Chem Soc, 2002
- A role for nitrilase  [ **NTHB** ]   [ **NTHA** ]  3 in the regulation of root morphology in sulphur starving Arabidopsis thaliana.  Ref: 11967096 Plant J, 2002
